# Demineralized Dentin Matrix Incorporated with rhBMP-2 Composite Graft for Treating Medication-Related Osteonecrosis of the Jaw

**DOI:** 10.3390/jcm13164830

**Published:** 2024-08-16

**Authors:** Jeong-Kui Ku, Jin-Won Choi, Seung-Min Song, Pil-Young Yun, In-Woong Um, Dae Ho Leem

**Affiliations:** 1 Department of Oral and Maxillofacial Surgery, Section of Dentistry, Seoul National University Bundang Hospital, 172, Dolma-ro, Bundang-gu, Seongnam-si 13605, Republic of Korea; kujk@snubh.org (J.-K.K.); 54845@snubh.org (J.-W.C.); smin3516@naver.com (S.-M.S.); pilyoung@snubh.org (P.-Y.Y.); 2 Department of Dentistry and Dental Research Institute, School of Dentistry, Seoul National University, Seoul 01811, Republic of Korea; 3 R&D Institute, Korea Tooth Bank, Seoul 06101, Republic of Korea; h-bmp@hanmail.net; 4 Department of Oral and Maxillofacial Surgery, School of Dentistry and Institute of Oral Bioscience, Research Institute of Clinical Medicine of Jeonbuk National University, Biomedical Research Institute of Jeonbuk National University Hospital, Jeonbuk National University, Jeonju 54907, Republic of Korea

**Keywords:** biofluorescence imaging system, bone regeneration, dental implants, demineralized dentin matrix, medication related osteonecrosis of the jaw

## Abstract

Medication-Related Osteonecrosis of the Jaw (MRONJ) is characterized by bone exposure in the oral and maxillofacial region for more than eight weeks in patients treated with anti-resorptive agents, immunosuppressants, or anti-angiogenic agents, without prior radiation therapy or metastatic disease to the jaws. Conservative treatments can control infection in mild cases, but surgical intervention is necessary for patients with severe symptoms. A 78-year-old female with a history of bisphosphonate treatment for osteoporosis presented with persistent pain, swelling, and malodor following implant placement in the upper right maxilla. SPECT/CT imaging revealed a high-risk hot spot in the right maxillary region. BIS-guided surgery using the Qray pen-C was performed, selectively removing red fluorescent bone tissue. The defect was grafted with HuBT incorporated with rhBMP-2. Postoperative follow-ups at 4, 7, and 14 months showed successful bone healing, transforming into a corticocancellous complex, and implant placement without MRONJ recurrence. Allogeneic demineralized dentin matrix (DDM) incorporated with rhBMP-2 demonstrates effective bone healing and implant placement following BIS-guided MRONJ surgery. This case supports the use of DDM/rhBMP-2 for tissue regeneration in MRONJ treatment, enabling successful prosthetic restoration without recurrence.

## 1. Introduction

Medication-Related Osteonecrosis of the Jaw (MRONJ) is defined as bone exposure in the oral and maxillofacial region lasting for more than eight weeks in patients who have received anti-resorptive agents alone or in combination with immunosuppressants or anti-angiogenic agents, without any history of radiation therapy or metastatic disease to the jaws [1]. Although the exact pathogenesis of MRONJ remains unclear, it is believed that the inhibition of osteoclastic activity prevents the removal of damaged bone following invasive treatment, leading to the accumulation of inflammatory factors and subsequent bone necrosis [2,3,4,5]. Conservative treatment aimed at infection control may be attempted in mild cases, but surgical intervention is necessary when patients present with clinical symptoms such as pain, swelling, or pus discharge [6]. Sequestrectomy and segmental resection can be an effective treatment at all stages of osteonecrosis of the jaw [7]. However, in 2024, Doub et al. reported a 33% recurrence rate post-surgery for drug-related or radiation-related osteonecrosis of the jaw, attributing recurrence to residual pathological lesions at the surgical margins [8]. Extensive resection may reduce the risk of recurrence but can result in significant defects, making prosthetic restoration, such as dental implants, challenging and significantly reducing the patient’s quality of life.

Recently, biofluorescence imaging system (BIS) devices have been explored for MRONJ surgeries. Among these, Qray (AIOBIO, Seoul, Republic of Korea) was developed to visualize and quantify porphyrin, a byproduct of late colony microbiomes, as red fluorescence distinct from tooth fluorescence [9,10,11]. Several studies have reported that infected bone tissue after grafting, endodontic infection, and MRONJ bone specimens exhibit fluorescence patterns similar to those of teeth [12,13,14]. In previous research on MRONJ specimens, tissues exhibiting red fluorescence showed bacterial invasion, while tissues without red fluorescence were identified as sclerotic with no osteocytes, suggesting that they could be left intact without pathological recurrence [12]. In 2024, a BIS-guided MRONJ surgery using Qray showed that selectively removing only bone tissue with red fluorescence resulted in new bone formation in the remaining bone, providing a basis for applying bone graft materials [13].

Demineralized dentin matrix (DDM) consists of highly cross-linked type I collagen with non-collagenous matrix-binding proteins such as bone morphogenetic proteins (BMPs), transforming growth factors, fibroblast growth factors, and insulin growth factors [15,16,17]. Allogeneic DDM (Allo-DDM) made from human dentin undergoes additional gamma irradiation to maintain osteoinductivity while minimizing antigenicity, commercialized as HuBT^®^ (Korea Tooth Bank, Seoul, Republic of Korea). Recently, Allo-DDM combined with rhBMP-2 (HuBT.BMP, Korea Tooth Bank, Seoul, Republic of Korea) has shown safer and more efficient clinical outcomes than Auto-DDM with rhBMP-2 (AutoDDM.BMP) [18]. This case report presents a patient successfully treated with Allo-DDM incorporated with rhBMP-2 and implant placement following MRONJ surgery.

## 2. Materials and Methods

This case report was approved by the Institutional Review Board of Jeonbuk National University Hospital (IRB No.: 2024-06-023) and conducted according to the principles of the Declaration of Helsinki. The IRB waived written informed consent due to the retrospective nature of the study.

**Figure 1:**
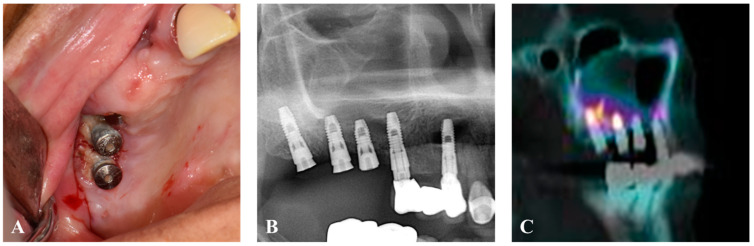
Preoperative images. (**A**) Bone exposure on right maxilla. (**B**) Preoperative panoramic x-ray. (**C**) Pre-operative SPECT/CT.

A 78-year-old female, in treatment for diabetes mellitus type 2, hypercholesterolemia, and hypertension, visited Jeonbuk National University Hospital (Jeonju-si, Republic of Korea) on 20 October 2022 and presented with persistent pain, swelling, and malodor after implant placement in the upper right maxilla. The patient had a history of osteoporosis treated with bisphosphonates (Fosamax 70 mg once weekly) since 2010, with bone exposure persisting for more than eight weeks. Surgery was planned for medication-related osteonecrosis of the jaw. Bone single-photon emission computed tomography/computed tomography (SPECT/CT) using technetium-99m hydroxymethylene diphosphonate revealed a high-risk hot spot from the right maxillary second molar to the right maxillary second premolar implants (Figure 1).

**Figure 2:**
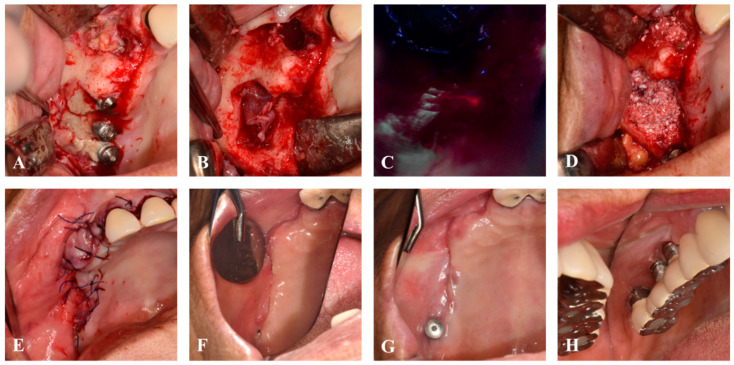
Intraoral images. (**A**) Necrotic bone was exposed on right maxilla. (**B**) Removal of sequestrum. (**C**) Red fluorescent bone was selectively removed by using Qray Pen-C. (**D**) HuBT incorporated with rhBMP-2 graft. (**E**) Primary closure. (**F**) Four months post-surgery. (**G**) Seven months post-surgery. (**H**) Fourteen months post-surgery.

Following the previously published BIS-guided MRONJ surgery protocol [13], necrotic bone around the implant was removed under normal light vision (Figure 2A,B). Using the Qray pen-C (AIOBIO, Seoul, Republic of Korea), the fluorescence pattern of the remaining bone was evaluated, and areas exhibiting red fluorescence were selectively excised (Figure 2C). After confirming the absence of red fluorescence at all margins, HuBT.BMP (Allo-DDM incorporated with rhBMP-2, Korea Tooth Bank, Seoul, Republic of Korea) was grafted into the bone defect (Figure 2D). The flap was advanced with a periosteal releasing incision for primary closure with 4-0 Vicryl (Ethicon, Johnson & Johnson international, New Brunswick, NJ, USA) (Figure 2E). Postoperatively, antibiotics and NSAIDs were prescribed for five days, with Sudafed 60 mg tid (pseudoephedrine, Samil Pharm., Seoul, Republic of Korea) and 0.12% hexamidine sol mouthwash used for two weeks, followed by stitch removal.

**Figure 3:**
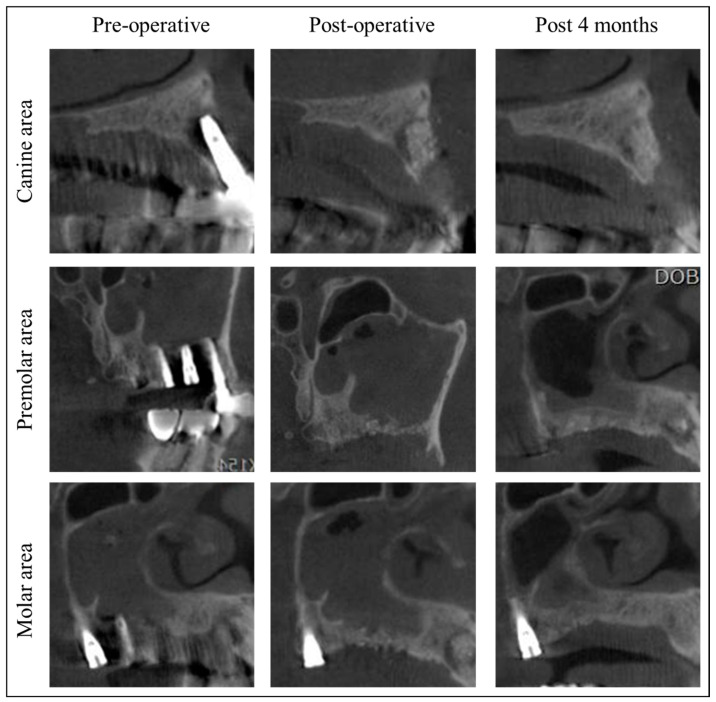
Cone beam computed tomography of this patient. At four months post-surgery, new bone formation with corticocancellous complex was identified at the location of the grafted Allo-DDM incorporated with rhBMP-2, with the subsidence of sinusitis.

**Figure 4:**
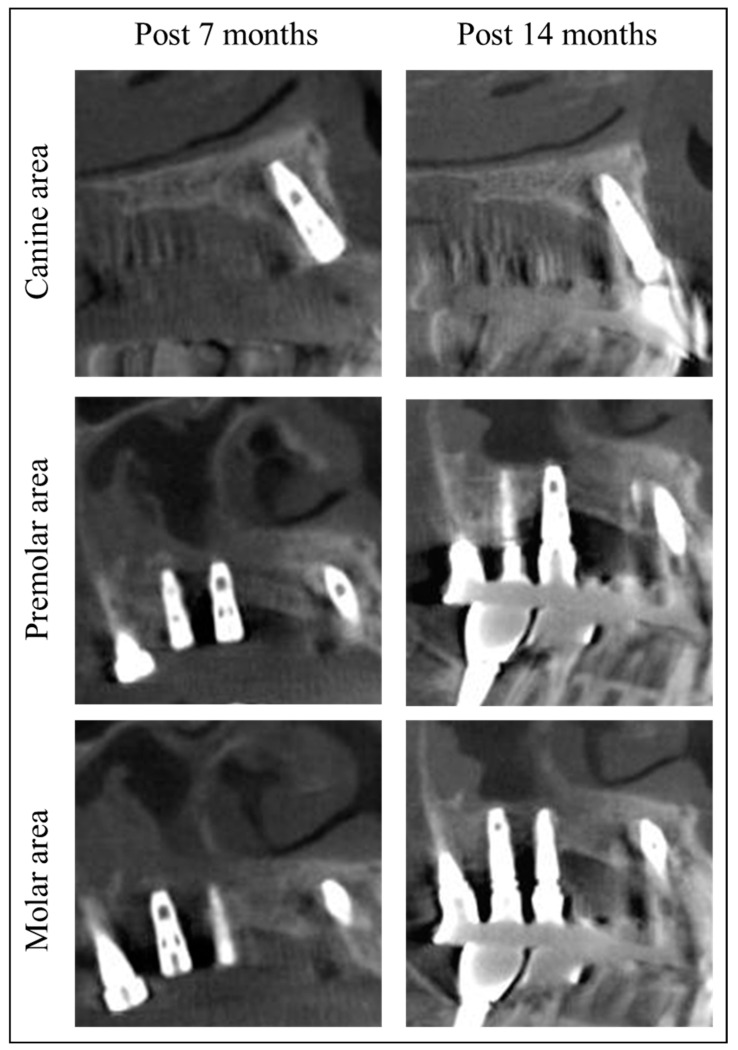
Cone beam computed tomography after of this patient. The regenerated new bone allowed for dental implantation without complications.

Since the patient’s residence was far from the hospital, she was instructed to have her stitches removed at a local dental clinic in 10 days. Post-surgery at 4 months, soft tissue and bone healing was successful without infection or MRONJ recurrence (Figure 2F and Figure 3). The patient stated that they underwent implant placement surgery again at a local dental clinic 5.5 months after the previous implant surgery, without any additional bone grafting surgery. At the 7-month follow-up, stable healing without bone resorption was observed, and the implant was placed at the local clinic (Figure 2G). Fourteen months post-surgery, there was no recurrence of MRONJ, and CBCT showed an improvement in sinusitis and the remodeling of the grafted bone into a cortico-cancellous complex, allowing for implant placement without additional bone grafting (Figure 4). The second maxillary molar implant site also showed no signs of MRONJ recurrence up to 14 months post-surgery.

**Figure 5:**
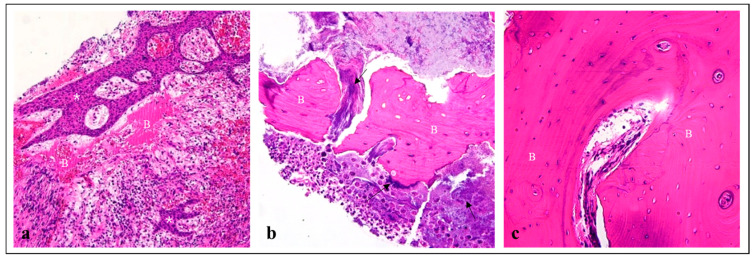
Hematoxylin and eosin staining of the specimens. (**a**) Sequestrum (×10), showing chronic inflammation (asterisk) and bone matrix destruction (B). (**b**) Red fluorescent bone tissue (×20), showing destructed bone matrix (B) with actinomycosis infection (Black arrow). (**c**) Non-red fluorescent bone tissue (×20), showing vital bone matrix (B) with osteocytes without matrix destruction or bacterial invasion.

Histological analysis revealed that both the sequestrum and red fluorescent bone showed the destruction of the bone matrix with actinomycosis infection, necessitating removal (Figure 5a,b). Non-red fluorescent bone tissue, removed for alveoloplasty due to its sharp edges, showed no pathological elements and was deemed normal bone tissue (Figure 5c). This finding aligns with previous research indicating that non-red fluorescent bone tissue need not be removed.

## 3. Discussion

This case report demonstrates successful bone healing and implant placement following BIS-guided MRONJ surgery. To the best of our knowledge, this is the first report of simultaneous bone grafting with DDM on the MRONJ surgical defect. Consistent with 2024 findings on non-red fluorescent bone in BIS-guided MRONJ surgery [13], this case confirmed that non-red fluorescent bone at the lesion margins is vital tissue. The grafted Allo-DDM incorporated with rhBMP-2 showed no complications. Both the existing and newly placed implants healed normally without MRONJ recurrence, as confirmed by CBCT and clinical examination.

The Qray, a BIS device, operates by detecting the red fluorescence of porphyrin, a metabolic product of oral microbiomes, under 420 nm high-intensity blue light [9]. Kim et al. analyzed the RGB values of fluorescence patterns in sequestra collected during MRONJ surgery using BIS techniques, categorizing them into non-red, hyper-red, and hypo-red fluorescence, each with distinct histological features [12]. Non-red fluorescence indicated sclerotic lamellar bone, hyper-red fluorescence indicated inflamed tissue with bacterial invasion and bone resorption, and hypo-red fluorescence indicated inflamed granulation tissue without bacterial colonies. While hypo-red tissues are easily distinguishable during surgery, non-red and hyper-red fluorescence have similar textures but different pathological implications. This background supports the finding of new bone formation in residual bones post-BIS-guided MRONJ surgery [13]. This study also confirmed that non-red fluorescent bone tissue collected from sharp bone margins during MRONJ surgery is intact bone tissue (Figure 4), suggesting that fluorescence-guided sequestrum resection can accurately determine surgical margins, removing pathological tissue while preserving healthy tissue.

In 2008, partially demineralized dentin matrix (AutoBT^®^, Korea Tooth Bank, Seoul, Republic of Korea) was developed by dehydration, degreasing, and ethylene oxide disinfection [19], and commercialized as a bone graft material that was covered by the national insurance system in the Republic of Korea. In 2017, AutoBT^®^ incorporated with rhBMP-2 (Auto-DDM/rhBMP-2; 0.2 mg/mL of rhBMP-2, Cowel Medi, Busan, Republic of Korea) was first used in socket preservation procedures, showing more active bone formation with the embedding of osteocytes compared to Auto-DDM alone [20]. Since 2019, Allo-DDM has been proven to be safe and efficient compared to its clinical outcomes with Auto-DDM, without any antigenicity and immunogenicity [18]. These results indicate that Allo-DDM can overcome the shortcomings of Auto-DDM, including an insufficient amount and delayed transplantation after extraction. The clinical outcomes of Allo-DDM incorporated with rhBMP-2 were comparable to those of Auto-DDM/rhBMP-2 in dental implants within the second year after functional loading.

For areas like MRONJ, where spontaneous bone formation is challenging [21,22], a bone-inductive agent capable of directly inducing the transformation of mesenchymal cells into osteoblasts is essential. Although there are reports that rhBMP-2 delivered on an absorbable collagen sponge (ACS) is effective for bone healing after MRONJ surgery [23], ACS has limitations regarding physical stability as a carrier in the in vivo environment. When implanted in the defect area post-surgery, this could lead to the undesirable local release of high doses of rhBMP-2, increasing the probability of adverse effects [24,25,26,27]. The combination of DDM with rhBMP-2, which has intrinsic osteoinductive properties, seems particularly effective. In this case, CBCT showed significant corticocancellous bone formation beyond the grafted area four months post-surgery, indicating the substantial osteoinductive capability of DDM/rhBMP-2 (Figure 4). However, due to personal circumstances, the patient received implant placement and prosthetic treatment at a local dental clinic, preventing the collection of data on bone formation and initial implant stability during surgery. Further research should include case series and longitudinal studies to evaluate bone quality and measure the volumetric bone formation through well-designed investigations involving a larger patient population.

## 4. Conclusions

Allogeneic demineralized dentin matrix incorporated with rhBMP-2 demonstrates successful bone healing and implant placement following BIS-guided MRONJ surgery. The newly formed bone in the MRONJ defect allowed for implant prosthetic restoration without MRONJ recurrence. Future studies should focus on volumetric change measurements using CBCT and histological analysis during implant surgery in bone regenerated with DDM/rhBMP-2 post-MRONJ surgery.

## Data Availability

Data are contained within the article.
